# A qualitative examination of the distribution strategies, access, and equitable uptake of the COVID-19 vaccines in Kenya: lessons for the next pandemic

**DOI:** 10.3389/fpubh.2025.1625970

**Published:** 2025-12-01

**Authors:** Stephen Mulupi, John Ele-Ojo Ataguba, Grace Njeri Muriithi, Daniel Malik Achala, Elizabeth Naa Adukwei Adote, Chinyere Ojiugo Mbachu, Senait Alemayehu Beshah, Chijioke Osinachi Nwosu, Elias Esfaw Zegeye, Vincent Okungu

**Affiliations:** 1 Taswira Health Research and Policy Consulting, Nairobi, Kenya; 2 African Health Economics and Policy Association (AfHEA), Accra, Ghana; 3 Department of Community Health Sciences, Max Rady College of Medicine, Rady Faculty of Health Sciences, University of Manitoba, Winnipeg, MB, Canada; 4 Partnership for Economic Policy (PEP), Nairobi, Kenya; 5 School of Health Systems and Public Health, University of Pretoria, Pretoria, South Africa; 6 Department of Community Medicine, University of Nigeria, Enugu, Nigeria; 7 Ethiopian Public Health Institute, Addis Ababa, Ethiopia; 8 Department of Economics and Finance, University of the Free State, Bloemfontein, South Africa; 9 Africa Centers for Disease Control and Prevention (Africa CDC), Division of Health Economics and Financing, Ababa, Ethiopia; 10 University of KwaZulu-Natal (UKZN), Economics Department, Durban, South Africa; 11 Department of Public and Global Health, University of Nairobi, Nairobi, Kenya

**Keywords:** COVID-19 vaccine, COVID-19 vaccine hesitancy, COVID-19 infection control, Kenya, vaccine equity

## Abstract

**Background:**

The WHO and international partners identified vaccination against the coronavirus as one of the important public health interventions in controlling Corona Virus Disease (COVID-19) infections. The COVID-19 vaccine uptake was however low and characterized by inequitable distribution and access in Kenya. This study aimed to examine in depth the causes of inequity in vaccine uptake to inform the future rollout and successful uptake of new vaccines.

**Methods:**

The study employed a qualitative approach involving in-depth key informant interviews with stakeholders across various aspects of COVID-19 healthcare, public health, vaccine distribution, and community engagement in Kenya (*n* = 32). Data were analyzed using the framework approach to allow for the identification, examination, and interpretation of patterns or themes emerging from the interview and review data.

**Findings:**

Centralized leadership provided by the Ministry of Health (MOH) utilized the existing distribution system, leveraging the Expanded Program on Immunization (EPI); enhancement of stakeholder collaboration and communication through government agencies, civil society, and community health workers was essential for the distribution of COVID-19 vaccines. Key barriers to vaccine uptake included organization and coordination of the vaccination rollout, socio-cultural barriers, and geographic challenges, particularly in rural areas, hindering vaccine access. Communication problems, particularly misinformation, were associated with public mistrust in vaccination efforts.

**Conclusion:**

Efforts to increase vaccine coverage should target organizational, social, political, and cultural norms to enhance access and uptake of quality vaccines. This study presents important lessons for health system adaptation, re-organization and preparations for future pandemics.

## Introduction

The Corona Virus Disease (COVID-19) pandemic had a significant impact all over the world, characterized by widespread deaths (1), socio-economic disruptions (2), and extreme stressors on health systems. Africa accounted for over 9 million infections and had more than 200,000 confirmed deaths by the first half of 2022. Specifically, Kenya had over 337,000 confirmed cases and upwards of 5,600 deaths (3). Global efforts to stop the spread of COVID-19 identified vaccination as a priority intervention in addition to existing infection control methods, i.e., sanitation and personal hygiene, control of people’s movement, including local curfews, targeted bans on international travel, social distancing regulations, and closure of businesses and schools (4, 5). Despite the rapid development and deployment of COVID-19 vaccines, most low-and-middle-income countries (LMICs) could not achieve at least 10% population coverage in the early stages of vaccine rollouts (6). As of December 14, 2020, after the first COVID-19 vaccine product was introduced, approximately 5.47 billion vaccine doses had been administered, and up to 56% of the global population was vaccinated with a complete primary series of COVID-19 vaccines. About 28% people received at least one booster dose of the vaccine.

The distribution of these vaccinations was uneven, characterized by the low vaccination rates in LMICs, where only 15% of the population received at least a single dose by the end of 2022 (7). The countries with the highest COVID-19 vaccine uptake rate included Puerto Rico (100%), Nicaragua (97%), Cayman Islands (95%), Cuba (95%), Portugal (95%), and Peru (93%). The countries with the worst uptake of the vaccine include Haiti (5%), Papua New Guinea (4%), and Yemen (4%). The contribution of vaccines in reducing COVID-19 prevalence has been immense. By early 2024, 135,000 cases were reported worldwide- a considerable drop compared to 2020 cases (7).

Early research in Kenya suggested that vaccine inequity was linked to a lack of transparency, poor coordination, and inconsistent messaging, which adversely affected specific population groups’ decisions on vaccine uptake, suggesting vaccine hesitancy. Vaccine hesitancy is a complex issue, meaning delays in taking vaccination even in situations where vaccination services are available (8). There were also rural–urban differences in access to trusted information channels about the vaccines, including radio, Television, and newspapers, as well as myths and misinformation to which communities did not have a meaningful response and were poorly equipped to address people’s concerns (9). Rajshekhar et al. reported significant variability in vaccine uptake among different age groups in Kenya’s urban slums, with older people more likely than younger ones to be vaccinated (10). A study by Anino et al. found that advanced age (58 to 98 years), combined with chronic disease, were associated with increased vaccine hesitancy (11). Long distances to vaccination centers were identified as a main factor for low vaccination uptake (12).

Global policy agenda in the post-pandemic era emphasize the need for preparedness, including increased local production capacities and access to medical products (13). The *“Equitable Access to COVID-19 Vaccines in Africa (ECOVA)”* project aims to reduce inequity in the manufacture and rollout of vaccines through research, advocacy, and capacity-building activities in Africa.

The main objective of this study was to examine in-depth the causes of inequity in COVID-19 vaccine access and uptake to inform the future rollout of new vaccines. Specific objectives were: to undertake country-level analysis of COVID-19 vaccine distribution and delivery mechanisms. To assess the required structures and mechanisms to ensure timely access to and administration of new vaccines, and to critically assess how non-governmental bodies can be engaged to improve COVID-19 and new vaccine delivery in Kenya and similar contexts. The lessons drawn from this study are essential for enhancing global preparedness for future pandemics.

## Methods

A case study design was adopted using Kenya as the case. Case study design is important for the evaluation of phenomena within their contexts (14). By understanding context, the study is positioned within boundaries of time, people, and place, and is useful to explain why certain observations were made. We adopted a constructivist approach (15) to explore the worldviews and realities of the key informants involved in the COVID-19 response. Understanding the perspectives of research participants is important in answering the ‘why?’ question and gaining insights about peoples’ preferences.

The study was conducted in Nairobi, Kenya’s capital, the seat of Government and focal point of COVID-19 control. Kenya runs a decentralized governance system, where the national government is mandated to steward policy design and capacity strengthening, while the 47 subnational (county governments) are mandated to translate national policy into service delivery. The counties provide the majority of health services from the community level to secondary referral hospitals.

Key informant interviews (*n* = 32) were conducted with key Ministry of Health, Kenya Medical Supplies Authority (KEMSA), and county governments (*n* = 24), non-governmental organizations (NGOs), and private sector representatives (*n* = 5), and healthcare workers (*n* = 3). The key informants were sampled purposively, based on their roles in developing or adopting policy for vaccination, planning and coordination, surveillance and pharmaco-vigilance, vaccines and drug supply chains management, community engagement, risk communication, financial management, and administrative roles. Key informants were identified and engaged through e-mails, phone calls, and physical meetings at their places of work. They were provided information about the study, and interview appointments were sought at least 2 weeks before the data collection. Data were collected between January and April 2025.

A team of two researchers with experience in qualitative research collected data through semi-structured interviews (Annex 1) in quiet private venues to enhance confidentiality and quality of the data collected. The topics explored in the discussions included existing vaccine delivery mechanisms, stakeholder engagement, challenges of distribution, demand creation, follow-up plans after vaccination, vulnerable populations, perceptions of equity, and recommendations for future preparedness for COVID-19 vaccines. Interviews were led by one researcher, and field notes were drafted by the second; discussions were audio recorded. Close to the end of the discussions, the second researcher was invited to seek clarification on emerging issues. This process took about 5–10 min and was essential to ensure comprehensive accounts. These interviews lasted 40 min on average.

Audio recordings were transferred immediately after the interviews to password-protected laptops, accessible only by authorized research team members. The research team debriefed to review key information emerging inductively from the interviews, which was iteratively embedded in subsequent interviews. Data were collected to theoretical saturation.

Data were analyzed using the framework approach, as described by Ritchie and Spencer (16). First, all the audios were transcribed verbatim, and the transcripts were reviewed and confirmed as accurate accounts of the interviews. The transcripts were read thoroughly, and personally identifying information was removed. Subsequently, a coding frame was developed based on the main themes, aligning with the key topics of the interview. Data were color-coded deductively on each transcript, based on broad interview topics, and a summary of key findings was developed, based on the themes in Word documents. Emerging issues were identified and coded inductively. Three research team members jointly reviewed the resultant document, theme by theme, and summarized key findings. Divergent opinions of the meanings were resolved through consensus. Data were triangulated across respondent groups to enhance trustworthiness and rigor. Verbatim excerpts have been anonymized to protect participants’ confidentiality. The initial findings were validated at a workshop attended by policymakers in Nairobi.

## Results

### Distribution of key informants by organizational affiliation

Table 1 outlines the distribution of key informants and organizational affiliations.

**Table 1:** Summary of interviewed respondents.

Category	Interviewees by organizational category			
	Government office staff	Partners	Frontline workers	Total no. of experts engaged
Surveillance and Pharmacovigilance	Ministry of Health (*n* = 4)	World Health Organization (WHO)		4
Planning and coordination	Ministry of Health (*n* = 1)	WHO	Health workers	1
Vaccine, cold chain, logistics & infrastructure	KEMSA (*n* = 2)			2
Vaccine, supplies	KEMSA (*n* = 3)	AMREF (*n* = 1) Equity Afia (*n* = 1)		5
Regulatory	Ministry of Health (*n* = 1)			1
Budgeting	Ministry of Finance			0
Pharmaceuticals Supply Agency	Ministry of Health (*n* = 2)		Health workers (*n* = 3)	5
Epi surveillance	Ministry of Health /County Department of Health (*n* = 2)		Health workers	2
Risk Communication and Community Engagement	County Department of Health (*n* = 2)	AMREF (*n* = 1) Equity Afia (*n* = 1)		4
Finance	Ministry of Health (*n* = 1)			1
Protection	Ministry of Health (*n* = 2)			2
Admin	Ministry of Health (*n* = 2)			2
Operational Research	Ministry of Health (*n* = 2)	AMREF (n = 1)		3
Total				**32**

Key themes emerging from the data analysis included: Ministry of Health (MOH) stewardship of the COVID-19 vaccine distribution and delivery; barriers to centralized coordination mechanisms; communication problems; organization of vaccine delivery; and geographical and cultural access barriers. Key recommendations for enhancing vaccine delivery in future situations, and suggestions for overall pandemic preparedness were also outlined.

### Ministry of health stewardship of vaccine distribution and delivery mechanisms

The interview narratives outlined aspects that were perceived to enable vaccine distribution and availability across the country. The majority of the respondents underscored the central role of the Ministry of Health (MOH) in stewarding the delivery of vaccines to communities through an inclusive approach. Kenya’s vaccine distribution operates under a centralized system managed by the MOH, leveraging the existing infrastructure of the Expanded Program on Immunization (EPI) and the supply networks of KEMSA. The MOH mobilized and engaged a wide range of actors, including subnational (county) governments; multilateral development partners (the WHO, UNICEF, and GAVI, the Vaccine Alliance), and humanitarian organizations, such as the Kenya Red Cross Society, *Médecins Sans Frontières* (MSF); civil society organizations, like the, African Medical Research Foundation (AMREF) and Christian Health Association of Kenya (CHAK). A key informant summarized the MOH stewardship as follows:

*In summary, the MOH leadership ensured a distinct chain of command in vaccine distribution and communication. This was essential in mobilizing key infrastructure in the health sector as well as in securing the support of local and external actors. (KI, 03).*

Strategic public-private partnerships (PPPs), with organizations such as Equity Afia Healthcare, Philips Distributors, and Mission for Essential Drugs and Supplies (MEDS), and access to their technical and infrastructural resources were essential for vaccine distribution, storage, administration, public awareness, and outreach programs to promote vaccine uptake and surveillance. For example, the Coca-Cola Project Last Mile, supply lines were used to enhance cold chain management and delivery to remote areas. Partnerships between public hospitals and private healthcare providers supported the expansion of vaccine access through community outreach programs. An added advantage of these PPPs was their ability to promote equitable vaccine distribution through deliberate efforts to include women, men, and marginalized groups in planning, decision-making, and outreach efforts. A respondent said:

*Some of the collaborative efforts between the state and the private sector have promoted gender equity by ensuring that women and men, as well as marginalized groups, are included in planning, decision-making, and outreach efforts, making vaccines more accessible to vulnerable populations. (KI, 23).*

**Figure 1:**
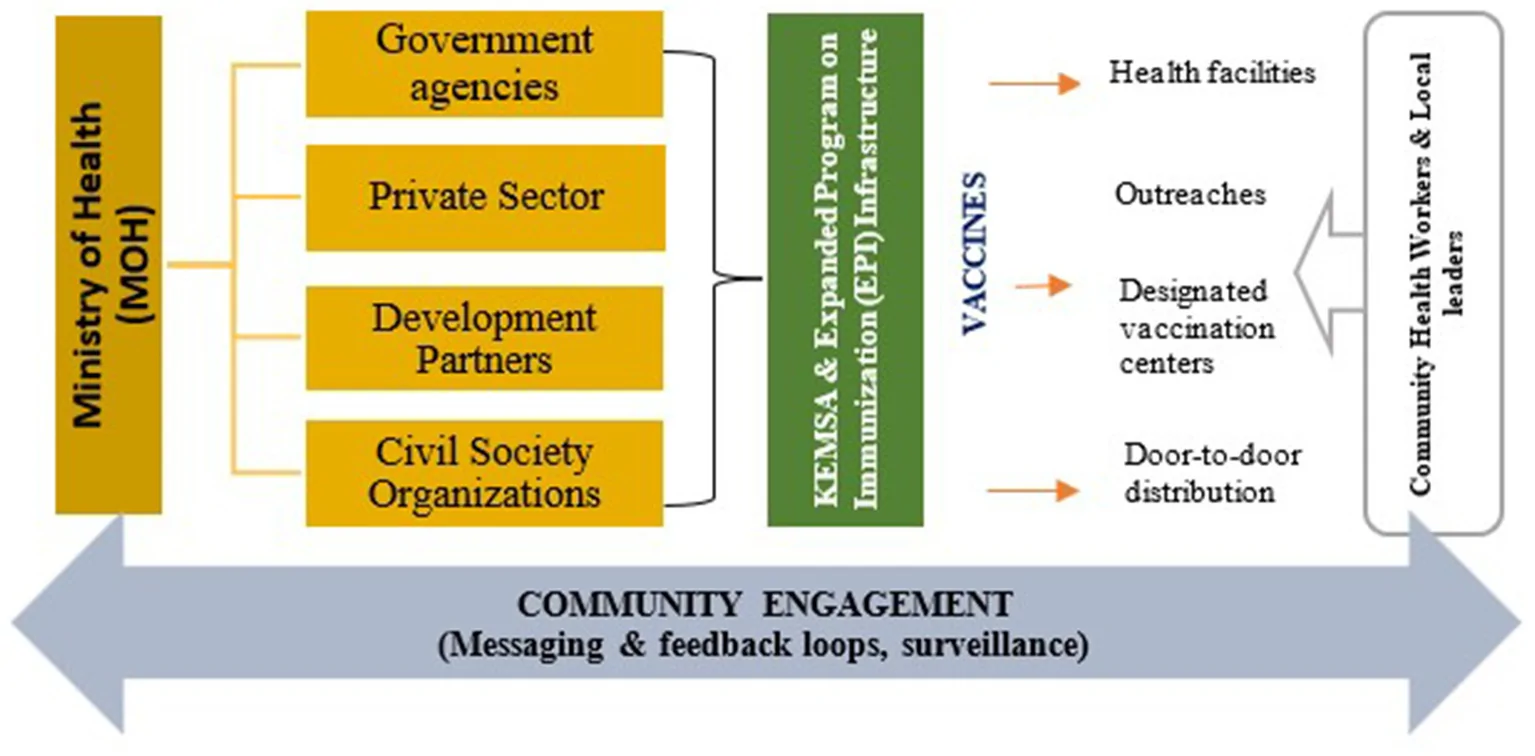
COVID-19 vaccine distribution infrastructure.

Additionally, the MOH developed a strategy for community engagement and sensitization on COVID-19 vaccination through community health workers, community leaders, and various media channels of nationwide coverage, including social media. This multipronged approach enhanced information flow and vaccine delivery systems (Figure 1).

According to key informants, the MOH vaccination campaign gave more priority to individuals and groups experiencing various forms of vulnerability, including victims of gender-based violence (GBV), people living with disabilities, refugees, children, and the older adults. These individuals and groups were identified for vaccination through community health systems. Several barriers were identified in the COVID-19 vaccine delivery processes in Kenya.

### Barriers associated with centralized coordination mechanisms

The majority of the study participants identified the delayed deliveries of vaccines from international suppliers based in the Global North countries as a significant impediment to national distribution mechanisms. Additionally, key informants described the hierarchical top-down, centralized ‘command structure’ from the MOH as having significantly affected the timeliness and equity of vaccine distribution. For example, it was reported that vaccine requests were initiated and centrally administered by senior officials at the MOH Headquarters, and KEMSA, the main government purchaser and supplier, could not independently process them. Consequently, KEMSA could not vary or rationalize the requests based on their assessment of demographics that need to be prioritized to ensure equitable access or timely procurement. Lack of reliable data was sometimes an impediment to timely decision-making. On delays to release vaccines, a respondent observed:

*Within the vaccine supply chain, there were two issues that stood out: vaccine hoarding by the global north that caused significant delays in getting the vaccines to the populations, and the bureaucracy at the MOH, which ensured long paperwork before KEMSA could get the authority to release the vaccines. (KI, 09).*

However, a divergent perspective was provided regarding KEMSA’s autonomy to purchase vaccines. Some county governments purchased their extra vaccine supplies from county budget allocations through KEMSA, and deficiencies were most likely in counties that relied on national government supplies only.

According to key informants from the MOH, government agencies must adhere to the *Public Procurement and Asset Disposal Act 2015,* which involves a complex procurement and asset disposal framework. They observed that procedural aspects in this framework implied longer decision-making and did not adequately respond to urgent procurement demands, such as in COVID-19. A key informant said:

*Yes, the PFM Act curbs corruption,[…] yes, we agree, but it is also quite rigid and creates a strict framework of procurement rules that prioritize accountability and transparency at the expense of interventions such as the COVID-19 vaccine distribution that required faster decision-making. (KI, 01).*

### Communication problems adversely affected COVID-19 vaccine uptake

The efforts by MOH to drive COVID-19 vaccination were affected by difficulties in communication to combat the rumors around the COVID-19 vaccines. According to interview narratives, the difficulty in addressing the spread of rumors was associated to a lack of trust in government COVID-19 initiatives. The rumors worsened because of the urgency to roll out new vaccines without allowing adequate time, public participation to synthesize and respond to concerns, and misinformation coming from various sources, particularly social media networks and religious leaders. For example, during the government vaccine mobilization efforts, there were widespread claims, associated with prominent religious leaders, that the COVID-19 vaccination initiative was a scheme for mass sterilization of women. An informant observed,

*Nowadays, information flows from all sources, each with its own version of stories. With COVID-19, the rumor started with a racial angle wherein the West had the intention of controlling the population of the Black race through COVID-19 and the vaccine that was designed to control it. The government was mistrusted to counter the Western influence because of its family planning efforts. (KI, 25).*

Other interview narratives identified key concerns about vaccine effectiveness, and risks of side effects, for example, male impotence, and long-term adverse effects. Some respondents observed that while the government communication strategy envisioned feedback loops among stakeholders in the COVID-19 vaccine distribution across the levels of health system, the feedback loops were sometimes dysfunctional, government response to misinformation perceived to be not timely, and in many instances, did not adequately address the misinformation from diverse sources. On dealing with misinformation, a key informant said,

*There was a lot of excitement about the information around the vaccines, and since we have freedom of speech, everyone was free to share their own versions of stories around the true intention of the vaccines…. This was a difficult situation to control, even though the government response itself was not properly coordinated.* (KI, 19).

### Inadequate preparation at the COVID-19 treatment centers and surveillance systems

Many designated COVID-19 vaccine-receiving centers were reported to be unprepared, impacting vaccine distribution and access. Key informants noted that such centers either had limited storage space or lacked the necessary infrastructure for vaccine cold storage, leading to delays in distribution and uptake. The lack of cold storage facilities led to another logistical challenge: the vaccines could not be offloaded from vehicles that had embedded cold storage systems, which were mostly hired from private logistics companies, rendering the logistics for distribution quite expensive. A key informant stated,

*In some cases, the vaccines could not be off-leaded because there is no cold storage, so they had to be taken to the next vaccination center with cold storage. This increased distance to vaccination centers for some people, thus affecting uptake.* (KI, 31).

Furthermore, some supplies arrived without the knowledge of administrators of the receiving centers, suggesting problems in coordination and communication between the MOH and implementing agencies on the ground. This meant that by the time the supplies arrived, there were neither proper preparations to receive and store the vaccines nor preparations to mobilize, train, and equip health workers to administer the vaccines to the community. Poor data systems complicated coordination; for example, delays in communicating vaccine stockouts affected new supply plans. A respondent clarified,

*In other instances, the distribution process was not properly planned with the technical teams on the ground. Imagine a track carrying vaccines arriving late at a facility, but there is no official to receive the products, and there is capacity at hand to start administering the vaccines. (KI,11).*

Respondents said Kenya’s COVID-19 surveillance system tracked vaccine distribution and adverse effects. However, some of the respondents stated that this surveillance system lacked gender and culture-sensitive approaches. They also added that, despite the existence of follow-up programs, these were not widespread, with limited targeted efforts for different genders. For instance, there were no specific programs in the community that followed up people after vaccination, yet vaccine effectiveness was generally tracked through national data on infection rates, hospitalizations, and adverse reactions.

### Geographical and cultural barriers to vaccine delivery and uptake

Interview narratives highlighted rural–urban disparities in vaccination uptake experience. According to some informants, geographical factors and other socio-cultural and systemic factors undermined COVID-19 vaccine access and uptake in remote rural settings. These included poor road networks and poor healthcare infrastructure in some rural areas. These factors added to existing layers of vulnerabilities in these areas, to impede uptake of vaccines, for example, language barriers for refugees, and accessibility problems for persons with disabilities, people living with chronic illnesses, and the older adults. Remote rural areas were also likely to have lower literacy rates and were perceived to be most vulnerable to widespread misinformation.

Most study respondents identified arid and semi-arid locations with poor transport infrastructure, as most affected by delays in vaccine delivery. Additionally, some respondents stated that these remote regions were particularly affected by a shortage of trained healthcare workers, unreliable cold chain infrastructure, and limited funding, further hindering vaccine distribution and administration. Interview narratives suggested that the lack of a decentralized cold-chain infrastructure to support the safe storage of vaccines, especially in geographically expansive but sparsely populated counties, compromised equitable access to the vaccines because of the long distance to vaccination centers. Inadequate access to cold storage was associated with vaccine wastage, though this was not quantified. Some rural remote regions, particularly in areas bordering other countries, also experienced widespread insecurity, including banditry and communal conflicts. A respondent noted,

*In the arid and semi-arid regions, there were many problems, including a poor road network, lack of health personnel, insecurity, and cultural issues, all made it especially difficult to access vaccines.* (KI, 18).

Remote rural locations were also associated with localized cultural beliefs. Religious restrictions and vaccine myths increased population vulnerability and negatively impacted vaccine distribution, delivery, and uptake. Several key informants associated vaccine refusal and hesitancy, especially among women, with patriarchal cultural norms, where household decision-making, including bodily autonomy and reproductive health issues, was largely influenced by men’s preferences. Women were also perceived to be more likely to observe religious teachings that prohibited vaccination and other forms of modern health interventions.

*In a patriarchal country such as ours, male decisions on whether to vaccinate or not carry the day. Add religion to that mix, and knowing that more women are involved in religious activities, if these sects state that they do not welcome vaccinations, this disproportionately impacts vaccine administration, especially to women and children.* (KI, 05).

Key informants from government agencies observed that men primarily oversaw logistics and regulatory processes, suggesting they had comparatively more access to information on COVID-19 vaccines than women. Instances of hesitancy among men were associated more with misinformation. A key informant explained:

*Although far-flung areas are challenging to access in terms of vaccine distribution due to poor transportation infrastructure, factors such as entrenched cultural beliefs, deeply ingrained religious beliefs, and myths and misinformation surrounding COVID-19, render these groups vulnerable to vaccine administration. This disproportionately renders women and children more vulnerable than men in the community.* (KI, 09).

To address the challenges in vaccine distribution and delivery, the MOH and partners devised strategies such as targeted outreaches, mobile clinics, and door-to-door campaigns, and adopted culturally sensitive messaging.

*When it comes to equitable access to vaccines, it is essential to note that gender disparities persist, with women having less decision-making power…. Therefore, while equity is measured by coverage, availability, and inclusion of vulnerable groups, financial, cultural, and logistical barriers remain critical to addressing vaccine equity.* (KI, 16).

### Recommendations to achieve equitable vaccine distribution and uptake in the future

In their reflections about priorities for enhancing vaccine delivery and uptake in case of future pandemics similar to COVID-19, the study participants suggested better ways of including the most vulnerable, gender-sensitive approaches, process and institutional changes, reorganization, specific legal reforms, and expanded use of public resources.

According to some, the most vulnerable people, particularly residents of remote and marginalized locations, could be better included through investments in mobile vaccination units in remote areas, outreach programs for marginalized groups, and tailored messaging to address cultural and gender-related barriers. This could be enhanced further by better engagement with communities through partnerships with community-based organizations and local media houses that communicated in the local dialect. A respondent explained,

*There is no better way to reach people with medical services than by getting the services as close to them as possible and utilizing their own resources to get to them. Community networks, local leaders, and local dialects can be effectively used to communicate and improve vaccination coverage and should be seriously considered in case of a new pandemic. (KI, 26).*

#### Better utilization of existing public resources and institutions

While acknowledging the existence of frameworks to address the present challenges in vaccine distribution, the respondents noted that the frameworks were inadequate and inefficient. Some participants identified missed opportunities, such as military resources and logistical capabilities, that could have been optimized, and recommended their use in future responses. Additionally, coordination with other government agencies, such as the Ministry of Interior and National Coordination, is required to support deliveries in areas experiencing increased insecurity. Additionally, respondents identified and recommended strengthening the National Disaster Management Unit (NDMU) to support vaccine management. The NDMU is an agency that already has an established command structure, an operational budget, and Standard Operating Procedures (SOPs) based on best practices. Enhanced government inter-agency coordination and collaboration at the national and county levels would mitigate supply-chain-related weaknesses, enhance commodity availability, particularly transportation, local storage solutions, and the capacity to administer vaccines. For instance, inter-governmental collaboration would improve commodity availability and identify appropriate local spaces for storage. A respondent stated,

*There is a* need *for inter-government agency coordination, with national and county governments working collaboratively and coordinatively to ensure availability of vaccine supply chain infrastructure to address weaknesses associated with lack of transport, storage, and capacities to administer the vaccines, among others. (KI, 01).*

#### Establishing a vaccines response technical working group

As a coordinating mechanism between national and county levels would enhance preparedness through engagement of thematic agencies in disease surveillance, vaccine distribution, and sustainability of vaccine deliveries and uptake during pandemics. The county-level TWG, for example, should focus on local-level vaccine administration preparedness and cold chain storage readiness. At the same time, the national-level TWG could help provide structures, policy direction, and nationwide oversight of vaccine distribution. Membership in the National-level TWG should include stakeholders such as the Council of Governors- the body representing subnational government leadership, MOH, the Ministry of Interior, private sector actors, and national and international humanitarian agencies. The county-level TWG should have local leadership. One respondent explained,

*There is a need to develop a framework that empowers both national and county governments to establish VRTWGs with the mandate to oversee thematic agencies’ preparedness on disease surveillance and vaccine distribution issues. This ensures* sustainability *of vaccine responses during pandemics such as the COVID-19 pandemic.* (KI, 22).

#### Legal reforms

Respondents also identified legal reforms, for example, specific amendments to the Public Procurement and Disposal Act, to allow for flexibility in procurement and increased responsiveness to emergencies, including the use of private sector services. An amendment that provides for direct procurement in emergencies, specifically bypassing multiple committee meetings, can improve efficiency in disaster response, including vaccine distribution.

#### Better mobilization of the private sector

The private sector actors can be mobilized better to support public emergencies and vaccine distribution, while incorporating gender-transformative processes through commercial incentives, including tax breaks and benefits, specific subsidies, public recognition, and public funding. The respondents also noted that Kenya is advancing local vaccine production capacity through initiatives such as establishing the Kenya BioVax Institute, which will support vaccine manufacturing. Such investments will help minimize Kenya’s over-reliance on foreign-led vaccine manufacturing, address monopoly and control by foreign governments, and enhance sustainability and timeliness in vaccine access. The interview narratives highlighted Kenyan government partnerships with global organizations such as Gavi and the Africa Centers for Disease Control (Africa CDC), in securing technology transfers and funding to enhance production capacity. A respondent said,

*The Kenya BioVax facility… is leading in vaccine innovations, a best practice which will wean the state from over-reliance on foreign-led vaccine manufacturing, addressing monopoly and control by foreign governments, and strengthening self-sustenance on vaccine access. Additionally, in partnership with global organizations such as GAVI and the Africa-CDC, the government is securing technology transfers and funding to enhance production capacity. (KI, 03).*

## Discussion

This study explored the underlying factors of COVID-19 vaccine distribution in Kenya, particularly access and equity. The findings outline aspects that enabled vaccine distribution under the stewardship of the MOH and systemic barriers, including institutional weaknesses, organizational challenges, communication difficulties, geographical, socio-economic, and cultural factors.

In detail, the centralized leadership and infrastructure utilization by the MOH played a pivotal role in leading the vaccine distribution efforts, leveraging an existing centralized structure that included the Expanded Program on Immunization (EPI) and KEMSA. The Ministry of Health, as a government agency, has legitimacy and convening power in national policy processes; therefore, was strategic in providing leadership in the pandemic response. The Kenyan constitution mandates that the national government design policies and support capacity strengthening at the subnational, devolved county governments. Legitimacy of a policy actor is essential in emotional appeal, securing support and buy-in of non-state actors, and sustaining policy initiatives (17).

The response by the partners in humanitarian organizations and private sector actors is expected. The COVID-19 pandemic significantly disrupted global economies, affecting businesses, education, and the daily lives of millions of people across the world (18). It could be argued that while the humanitarian organizations were responding to natural calamities affecting people as part of their core business, the private-for-profit actors’ responses suggest a collective acknowledgement of a threat to business survival (19). Engagement of the private sector actors also epitomizes the concept of “from government to governance” and lately, meta-governance (20)The fact is that governments no longer dominate policies but are increasingly reliant on technologies, specialist resources, geographical knowledge, technical know-how, and the goodwill of the private sector actors. The COVID-19 response required contextual, tailored responses. The importance of such partnerships has been highlighted by studies on COVID-19 control and prevention in various countries (21, 22).

Difficulties experienced due to existing government structures and centralized control have been identified in many other contexts worldwide. Jansenn et al. have expounded governments’ responses using a lens of agility and adaptability (23). According to their reflections, “successful adaptive governance calls for both decision speed and sound analysis, for both centralized and decentralized decision-making.” Whereas agility relates to speed in response, adaptivity, on the other hand, acknowledges that systemic changes at multiple levels of the health systems can sometimes take more time as multiple institutions struggle to align to new realities. This may create conflict between the need for speed, as in the case of procurement, and institutions working within the confines of existing legal and regulatory dictates. Similar observations were made by Torre and Storer (24) and Goel et al. (25) who recommend the urgent need to address bureaucratic hindrances in emergency situations.

Trust in government initiatives emerged as an important hindrance to the COVID-19 uptake, despite an already existing culture and practice of routine immunization and infrastructure in Kenya. Gilson defines trust as “a voluntary action based on expectations of how others will behave in relation to yourself in the future,” underscoring the relational nature of health systems as social institutions (26). When these expectations are unmet, this may generate negative outcomes, since there is an element of risk relating to motives, intentions, or actions. Trust is viewed as a strategic social capital and is vital in facilitating collective actions, particularly in circumstances that require cooperation to achieve a common societal goal (26).

This study suggests that certain aspects of the COVID-19 vaccination rollout triggered distrust. The lack of timely and accurate information dissemination around vaccine efficacy and side effects led to heightened skepticism, reflecting trends observed in other COVID-19 vaccination campaigns globally (27–31). This study established that people had concerns about the urgency of government getting everyone vaccinated amidst a burgeoning “infodemic,” the fast spread of misinformation that characterized public discourses during the COVID-19 response.

Effective crisis and risk communication is proposed as an important social determinant of health (32). There is a need for careful review of the broader implications of distrust in health systems interventions for public policies in the future, particularly in contexts that need urgent public co-operation with government initiatives, and a rapidly expanding social media knowledge exchange ecosystem. There is scope for future studies to examine why the public may distrust government initiatives on vaccines. There is also a need for contextual understanding and acknowledgement of widespread ethical concerns about vaccination (33) including historical issues of unethical vaccine research, particularly in poor contexts (34). COVID-19 vaccine anxieties have been widely reported in other international contexts, so the Kenyan experience is not an isolated issue (35, 36).

The study underscored the difficulties in reaching vulnerable populations, particularly in remote rural and arid regions where roads are impassable in some sections and healthcare infrastructure, including cold storage, are lacking. Transport and storage challenges hindered effective vaccine distribution, echoing findings from other studies that have examined the impact of geographic location on healthcare access (37–40). Language barriers and accessibility problems for persons with disabilities further complicate the situation, underscoring the need for inclusive healthcare strategies that consider diverse community needs (41, 42).

The findings suggest gendered disparities in access to information about the vaccine and decision-making for vaccine uptake. We add to the voices that champion effective community engagement, in the development of intervention packages that are responsive to socio-cultural needs, and worldviews (41, 43–45). We particularly focus on gendered power relations that may work to undermine individual autonomy, religious authorities that shape people’s worldviews (46, 47). Women are disadvantaged in contexts of patriarchy, where males dominate and shape decision-making for many socio-economic activities, including access to healthcare (48–51). This finding is supported by previous research indicating that gender dynamics can heavily influence health-related decisions (52–57). Other studies in Africa have established similar findings (58, 59).

In the aftermath of the COVID-19 pandemic, various research and policy communities around the world have sustained conversations toward reimagining health systems (60) to enhance future readiness post-pandemic. This study contributes to this discourse by highlighting nuanced imaginations about reorganizing the health system and improving preparedness in case a similar calamity strikes. Policy makers’ interests and priorities significantly influence policy design and translation processes. The recommendations for using readily available public resources are important in minimizing duplication and resultant wastage of scarce resources and time in responding to pandemics. Suggestions to enhance local vaccine production capacities, to safeguard against vaccine access inequities that manifested globally during the pandemic, align with the broader policy discussions for African governments to cut over-reliance on foreigners (61), through Africa-based research and development (62).

### Strengths and limitations

This study involved people in strategic decision-making positions in policy and delivery of COVID-19 vaccines. It therefore provides important and policy-amenable insights and issues that emerge as priorities for these policy actors. The main limitation of the study is the exclusion of the voices of vaccine users. Numerous studies have explored this issue, however, and therefore, these findings complement those perspectives. Future studies should consider measuring the costs of inequitable distribution of vaccines, delays, and destruction of vaccine due to infrastructural constraints.

## Conclusion

Whereas the Kenyan government demonstrated significant political goodwill in driving up vaccination for COVID-19, several organizational, socio-cultural, and systemic factors undermined this quest. The perspectives of the policy actors provide important insights and consideration for strengthening preparedness of the Kenyan system, for future pandemics.

## Data Availability

The raw data supporting the conclusions of this article will be made available by the authors, without undue reservation.
