# Correction: A qualitative examination of the distribution strategies, access, and equitable uptake of the COVID-19 vaccines in Kenya: lessons for the next pandemic

**DOI:** 10.3389/fpubh.2026.1974839

**Published:** 2026-09-01

**Authors:** Frontiers Production Office

**Affiliations:** Frontiers Media SA, Lausanne, Switzerland

**Keywords:** COVID-19 vaccine, COVID-19 vaccine hesitancy, COVID-19 infection control, Kenya, vaccine equity

The **Funding** of the article processing charges was erroneously omitted. The following text has now been added to the **Funding** statement:

“The author(s) declared that financial support was received for this work and/or its publication. The article processing charges for this article were funded by the International Development Research Centre (IDRC). The funder had no involvement in the handling, peer review, or editorial decision for this article.”

The original version of this article has been updated.

