# Supplementary material for: A qualitative examination of the distribution strategies, access, and equitable uptake of the COVID-19 vaccines in Kenya: lessons for the next pandemic
Source: Front Public Health. 2025 Dec 1;13:1625970. doi: 10.3389/fpubh.2025.1625970 (PMC12702695; doi:10.3389/fpubh.2025.1625970)
Supplement: Supplementary file 1 [file Supplementary_file_1.docx]

# ANNEX 1: Tool 1: Interview topic guide for key informants’ interview (KII)

**Section 1: General information**

| Interview type: | Key Informant Interview (KII) |
| --- | --- |
| Country |  |
| Level of the interview: |  |
| Affiliation and role of interviewee: |  |
| Location of Interview: |  |
| Respondent number (ID): |  |
| Name of Interviewer: |  |
| Gender of interviewer: |  |
| Gender of note taker (if applicable): |  |
| Date of interview: |  |
| Duration of the interview | Starting time:  Ending Time:  Duration: /____/_____/ minutes |
| General observations/comments  e.g., interview cut short, noisy environment, etc. |  |

**Section 2: Background information**

| No | Characteristics | Responses |
| --- | --- | --- |
| 1 | Age: |  |
| 2 | Gender: |  |
| 3 | Highest level of your educational qualification: |  |
| 4 | Brief description of their role in the organization |  |
| 5 | Years of service in COVID-19 vaccination-related works |  |
| 6 | Years of service in management or leadership positions in the public health system |  |

(No item should be left unanswered; say none if there are no comments. Use additional paper as needed).

Interviewer: __________________________

| **Qualitative topic guide to assess COVID-19 vaccine distribution and delivery** |
| --- |
| **Section 1: Introduction**   1. What role did your organization/institution play in the COVID-19 vaccine?   What is your role and responsibility in this?  **Section 2: Understanding Existing Mechanisms**   1. Can you describe Kenya's vaccine distribution and delivery mechanisms for COVID-19 vaccines? (Probes: Who is involved and in what capacity and influence? What proportion of them are men/women/ identities? Who [in terms of gender identity] is leading what – surveillance, planning & coordination, cold chain system, vaccine supplies, regulation, RCCE, etc.?) 2. What challenges or gaps have you observed in Kenya's vaccine distribution and delivery mechanisms? (Probes: Challenges related to societal and cultural norms (including gender norms), values and beliefs   **Probe:** Especially concerning reaching out to vulnerable populations?  How do you see the availability of resources, including human resources?   1. What does the COVID-19 vaccine supply chain look like? (Probe as 2) What are the challenges that were encountered in the supply chain? (Probe as 3) 2. What is your opinion about the demand creation work in the country? (Probes: are demand creation approaches to gender and culture-sensitive, and how? Are the messages designed for the various groups of women/men/other gender identities?   5b) What is your opinion about the surveillance work in the country? (Probes: are surveillance approaches to gender and culture-sensitive, and how do they appeal to various groups of women/men/other gender identities?  Prop: In your community, are there any programs that follow up with people after they get vaccinated for COVID-19? (Probes: Programs that target women, men, and people of other gender identities)  Prop: Do you know if there are ways to track how well the vaccines are working to prevent serious illness from COVID-19?  **Section 3: Vulnerable Populations and Equity**   1. Who, in your opinion, are the most vulnerable populations in the country/ Kenya concerning access to and administration of COVID-19 vaccines? (**Probes**: What groups of women/men/other gender identities had less access to COVID-19 vaccines, and why [explore **individual-level factors** such as age, marital status, head of household (decision making), education, literacy, employment, occupation, geographic location, access to media, ethnicity, tribe, religion, health status – co-morbidity, disability, mental health status, etc.; **societal and cultural factors** such as gender norms, values, beliefs, myths/misconceptions, etc.; **environmental factors** such as displacement/refugee status, institutionalization (prisoners, etc.), conflicts, etc. 2. What is your opinion regarding equity in vaccine distribution? (**Probe**: how is equity defined/operationalized at the various levels of the vaccine distribution system? What parameters are used? Any of them related to gender and other social identities) 3. How do you think these vulnerable populations can be identified and reached effectively for vaccination? (**Probe**: Vulnerable groups of women/men/other gender identities as in 6)   **Section 4: Required Structures and Mechanisms**   1. What are the country-level efforts to start domestic vaccine manufacturing?   **Probe**: These national efforts include attracting foreign investments to set up vaccine manufacturing companies.  As part of these efforts, which COVID-19 vaccines are being considered for production? (**Probe**: Any considerations of safety of vaccines for women of reproductive age (15 – 49)?   1. What additional structures or mechanisms are necessary to ensure timely access to and administration of vaccines in the country/ Kenya, particularly for vulnerable groups of women/men/other gender identities (**Probe** for vulnerable groups identified in 6)?   (Probe structures for organization of the health system; gender desk; communication; integration & coordination of distribution mechanisms [avoid competing initiatives; integrate health services, for example, antenatal])  **Section 5: Stakeholder Involvement**   1. How can the significant stakeholders be better engaged in a coordinated action plan, including advocacy to address inequities in access to vaccines in your country?   **Probe:** How important is it to involve various stakeholders, such as government agencies, healthcare providers, community organizations, and international partners, in improving vaccine distribution and delivery mechanisms? Probe for gender representation in stakeholder engagement.  **Probe:** Involvement of representatives of vulnerable groups of women/men/other gender identities. Engagement of men.   1. What specific roles do you think these stakeholders should play in ensuring equitable vaccine distribution in the country/ Kenya? 2. Do you have experience or expertise in engaging civil society organizations and the private sector in public-private partnerships for healthcare initiatives in the country/ Kenya? Can you describe your experience? (Probe whether there is a gender leaning - focused on men/ women/other gender identities)   **Probe:** Mention specific private companies involved and their contributions to improving vaccine logistics and cold chain management.  **Probe**: What are the key advantages of public-private partnerships in improving vaccine delivery and access in Kenya? (Probe: What is the benefit of improving access to vulnerable women/men/other gender identities, and how?)   1. Can you provide examples of successful public-private partnerships in the healthcare sector, particularly regarding vaccine distribution, that can serve as models for the country/ Kenya? (Probe: partnerships that promote gender representation in decision-making and implementation, gender equity in access to vaccines, engagement of vulnerable women/men/other gender identities; approaches that promote gender-responsive/transformative health programming) 2. How can the private sector, including pharmaceutical companies and healthcare providers, be effectively engaged in supporting equitable vaccine distribution in the country/ Kenya?   **Probe:** What incentives or strategies can encourage private sector involvement in addressing vaccine access disparities, including gender disparities?   1. What potential challenges or concerns do you foresee in engaging civil society organizations, non-governmental bodies, and the private sector in improving vaccine delivery in the country/ Kenya?   **Probe:** How can these challenges be mitigated or addressed to ensure the success of such collaborations?  **Section 6: Impact and Evaluation**   1. How would you assess the impact of improved vaccine distribution and delivery mechanisms on the overall public health situation in the country/ Kenya? (Probe for impact on men and women)   Probe: Reduced Disease Burden   1. What key performance indicators or metrics should be used to evaluate the success of these mechanisms? (Probe: sex-disaggregated data, gender-specific indicators, indicators for assessing success among vulnerable groups of women/men/other gender identities as in 6)   **Section 7: Recommendations**   1. Do you have any specific recommendations for policymakers and healthcare authorities in the country/ Kenya to enhance vaccine distribution and delivery mechanisms for COVID-19 vaccines, with a focus on vulnerable groups of women/men/people of other identities?   **Section 8: Additional Comments**   1. Do you want to share any other information or insights regarding this topic? |
